# Evaluating the Effect of Varying the Metal Precursor in the Colloidal Synthesis of MoSe_2_ Nanomaterials and Their Application as Electrodes in the Hydrogen Evolution Reaction

**DOI:** 10.3390/nano10091786

**Published:** 2020-09-09

**Authors:** Zakhele Ndala, Ndivhuwo Shumbula, Siyabonga Nkabinde, Tshwarela Kolokoto, Obakeng Nchoe, Poslet Shumbula, Zikhona N. Tetana, Ella C. Linganiso, Siziwe S. Gqoba, Nosipho Moloto

**Affiliations:** 1 Molecular Sciences Institute, School of Chemistry, University of the Witwatersrand, Private Bag 3, Wits 2050, South Africa; 491384@students.wits.ac.za (Z.N.); 677753@students.wits.ac.za (N.S.); 564058@students.wits.ac.za (S.N.); 670850@students.wits.ac.za (T.K.); 2291563@students.wits.ac.za (O.N.); Zikhona.Tetana@wits.ac.za (Z.N.T.); Cebisa.Linganiso@wits.ac.za (E.C.L.); 2 Department of Chemistry, University of Limpopo Private Bag x1106, Sovenga 0727, South Africa; poslet.shumbula@ul.ac.za; 3 DST/NRF Centre of Excellence in Strong Materials, University of the Witwatersrand, Private Bag 3, Wits 2050, South Africa; 4 Microscopy and Microanalysis Unit, University of the Witwatersrand, Private Bag 3, Johannesburg, Wits 2050, South Africa

**Keywords:** molybdenum diselenide, colloidal synthesis, HER

## Abstract

Herein we report on the use of different metal precursors in the synthesis of MoSe_2_ nanomaterials in order to control their morphology. The use of Mo(CO)_6_ as the metal precursor resulted in the formation of wrinkled few-layer nanosheets, while the use of H_2_MoO_4_ as the metal precursor resulted in the formation of nanoflowers. To investigate the effect of the morphologies on their performance as catalysts in the hydrogen evolution reaction, electrochemical characterization was done using linear sweep voltammetry (LSV), cyclic voltammetry (CV), and electrical impedance spectroscopy (EIS). The MoSe_2_ nanoflowers were found to have superior electrochemical performance towards the hydrogen evolution reaction with a lower Tafel slope, on-set potential, and overpotential at 10 mA/cm^2^ compared to the wrinkled few-layer nanosheets. This was found to be due to the higher effective electrochemical surface area of the nanoflowers compared to the nanosheets which suggests a higher number of exposed edge sites in the nanoflowers.

## 1. Introduction

2D layered materials such as MoSe_2_ are quickly becoming some of the most exciting materials, with interesting properties that have put them at the forefront for use in various applications and technologies including energy storage and generation devices, field-effect transistors and biosensors [1,2,3]. MoSe_2_ is a layered transition metal dichalcogenide (TMD) material where each layer is comprised of atom-thin layers of transition metal sandwiched between two chalcogen layers. Monolayer and few-layer MoSe_2_ nanomaterials have been shown to have interesting properties that can be used in various applications [4,5,6]. Colloidal synthesis is a wet-chemical technique that has been identified to have the potential to synthesize TMDs such as MoSe_2_ in high yields with control over the size, morphology and layer thickness which would, in turn, control their properties [7,8,9]. This control is achieved through the modification of the reaction parameters such as the temperature, chemical reagents, solvent/surfactant, and reagent concentration. 

Unfortunately, achieving this level of control on the properties of TMD nanomaterials is not a small task and is currently a significant challenge in the synthesis of TMDs using colloidal synthetic techniques. There has been a significant effort to investigate how the reaction parameters can be used to control some properties of the TMD nanomaterials [10,11,12]. Much of the work on the effect of precursors on the synthesis of MoSe_2_ nanomaterials has focused on the effect of the selenium precursor and largely ignored is the effect of the metal precursor [13,14,15]. The effect of the metal precursor choice in colloidal syntheses has been shown to be crucial in controlling the size, morphology, and phase of nanomaterials [16,17,18,19]. This work investigates the unexplored effect of two different metal precursors on the morphology of MoSe_2_ nanomaterials. In this work molybdenum hexacarbonyl and molybdic acid were used to evaluate the effect of these different metal precursors on the morphology of MoSe_2_ nanomaterials. 

To demonstrate how important a difference in the morphology of the MoSe_2_ nanomaterials can have on any application, the electrochemical properties of the nanomaterials were evaluated for application of the nanomaterials in the hydrogen evolution reaction (HER). The hydrogen evolution reaction is an electrocatalytic technique that is used in the production of hydrogen gas. This process is important because it provides a way to produce hydrogen which is a clean and efficient energy carrier in an eco-friendly manner [20]. MoSe_2_ has been identified as a potentially cost-effective alternative to platinum which is the current catalyst used for the HER [21,22]. The active sites for the HER in MoSe_2_ have been identified as the edge sites [22,23]. Thus, finding a morphology that maximizes the exposure of these edge sites in MoSe_2_ is crucial. This work shows how changing the morphology of the MoSe_2_ nanomaterials using the metal precursors influences the catalytic activity of the nanomaterials. 

## 2. Experimental

### 2.1. Chemicals

Molybdenum hexacarbonyl (Mo(CO)_6_, 98%, Sigma-Aldrich, Modderfontein, South Africa), molybdic acid (H_2_MoO_4_, 85%, Sigma-Aldrich), selenourea (CH_4_N_2_Se, 98%, Sigma-Aldrich), oleylamine (OAm, 70%, Sigma-Aldrich), toluene (anhydrous, 99.8%, Sigma-Aldrich) and ethanol (absolute, ≥99.8%, Sigma-Aldrich) were used as received without further purification.

### 2.2. Synthesis of MoSe_2_ Nanomaterials

#### 2.2.1. Synthesis of MoSe_2_ Wrinkled Few-Layer Nanosheets

A three-neck round bottom flask was filled with 20 mL of OAm and degassed in N_2_ for 20 min. The temperature was increased to 50 °C and 0.362 g (2.49 mmol) of selenourea was added to the reaction mixture and stirred for 10 min. At 220 °C, the selenourea decomposes to H_2_Se and a carboamide (C(NH)_2_), this is shown in the TGA profile in Figure S1. After selenourea decomposes the colour of the mixture changed from black to a dark orange colour. Molybdenum hexacarbonyl (0.212 g, 0.802 mmol)) was dissolved in the OAm. The mixture of molybdenum hexacarbonyl and OAm was then injected into the selenourea mixture at 300 °C. The reaction mixture turned black immediately. The reaction was allowed to run for 120 min. The black powder product was washed and collected by centrifugation using toluene and ethanol.

#### 2.2.2. Synthesis of MoSe_2_ Nanoflowers

In a three-neck round bottom flask 20 mL of OAm was added, the solvent was degassed for 20 min under nitrogen. The temperature was increased to 50 °C and 0.4 g (3.0 mmol) of selenourea was added to the reaction mixture and stirred for 10 min. About 0.2 g (0.15 mmol) of molybdic acid was added once the reaction temperature reached 300 °C. The reaction was allowed to run for 120 min, after which the product was collected and washed with toluene and ethanol by centrifugation.

### 2.3. Characterization Techniques

The phase purity, crystallinity and preferred crystal orientation of the products were examined by using PXRD on a MeasSrv (D2-205530)/D2-205530 diffractometer (Bruker, Billerica, MA, USA) using secondary graphite monochromated CoKα radiation (λ 1.78897 Å) at 30 kV/30 mA. Measurements were taken using a glancing angle of incidence detector at an angle of 2°, for 2θ values over 10–90° in steps of 0.026° with a step time of 37 s and at a temperature of 25 °C. Raman spectroscopy experiments were performed on a J-Y T64000 micro-Raman spectrometer (Horiba Jobin-Yvon, Ltd., Stanmore, UK) equipped with a liquid nitrogen cooled charge-coupled device detector. All samples were measured after excitation with a laser wavelength of 514.5 nm. The particle sizes and morphologies were determined by the transmission electron microscopy (TEM) carried out on a Technai T12 TEM microscope (FEI, Hillsboro, OR, USA) operated at an acceleration voltage of 200 kV with a beam spot size of 20–100 nm in TEM mode. The HRTEM images were obtained from a JEM-2100 microscope (JEOL, Akishima, Tokyo, Japan) equipped with a LAB6 filament and an EDS detector, operated at 200 kV. SEM images were obtained with a high-resolution FEI Nova Nanolab 600 instrument at 30 kV (FEI, Hillsboro, OR, USA). The total surface area and pore volume of the nanomaterials were ascertained using a Micromeritics TriStar Surface Area and Porosity Analyzer (Micromeritics Instrument Corp., Norcross, GA, USA). The electrochemical measurements were carried out on a BASi epsilon E2 (1231) (West Lafayette, IN, USA). All measurements were carried out in 0.5 M H_2_SO_4_ using a three-electrode system. A Ag/AgCl electrode was used as the reference electrode, platinum wire was used as the counter electrode. A modified glassy carbon electrode with 3 mm diameter was used as the working electrode. The ink or fresh dispersion of the sample was prepared by dispersing 5 mg of the MoSe_2_ nanomaterials with 0.5 mg carbon black and 40 µL of Nafion solution (5 wt%) in a 1 mL mixture of water and isopropanol at a 3:1 ratio. The solution was then sonicated for 30 min and 5 µL of the ink was drop-casted onto the glassy carbon electrode. The electrode was allowed to dry at room temperature. The linear sweep voltammograms were obtained at a scan rate of 2 mV/s and the cyclic voltammograms were obtained at a potential range of 0.2–0.6 V vs RHE. A 20% Pt/C catalyst was used for comparison. The impedance spectroscopy studies were conducted on a SP 300 system (Biologic, Seyssinet-Pariset, France). The measurements were done at a potential of −200 mV vs RHE and it was done at a frequency range between 0.01 Hz and 100 kHz.

## 3. Results and Discussion

### 3.1. Structural Characterisation

**Figure 1:**
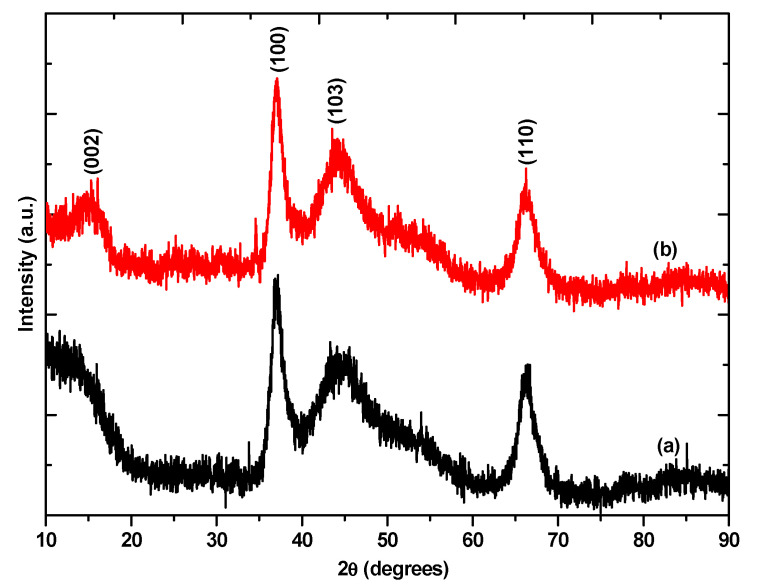
Powder X-ray diffraction patterns of (**a**) MoSe_2_ nanosheets (**b**) MoSe_2_ nanoflowers.

Powder X-ray diffraction (PXRD) was used to confirm the synthesis of MoSe_2_ and to also determine the crystallinity, phase and composition of the nanomaterials. The PXRD confirms the formation of 2H hexagonal MoSe_2_ nanomaterials (JCPDS card no: 03-065-3999). The reflection peaks located at 14.63°, 37.06°, 44.44°, and 66.02°, corresponding to the lattice planes (002), (100), (103), and (110) respectively. The broad nature of the peaks as shown in Figure 1 is indicative of nanostructured materials. 

**Figure 2:**
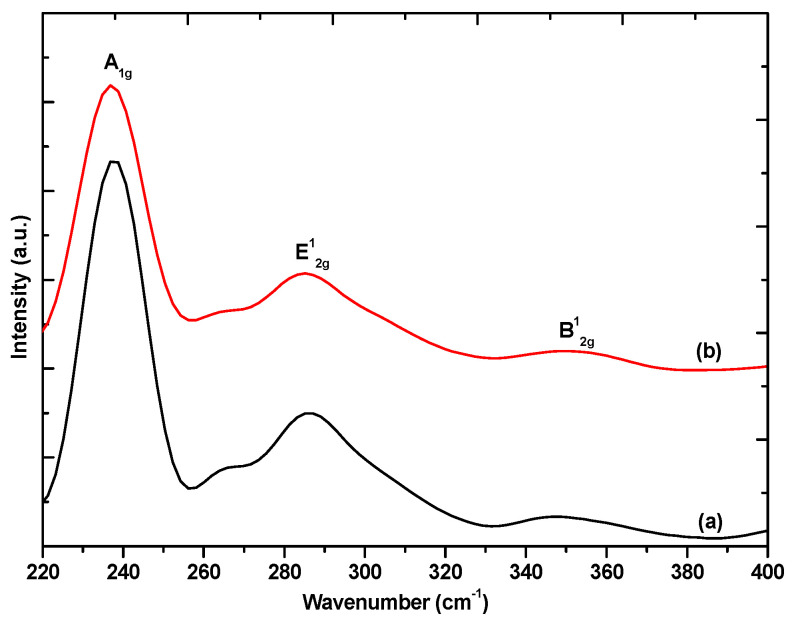
Raman spectra of (**a**) MoSe_2_ nanosheets and (**b**) MoSe_2_ nanoflowers.

There are no peaks not belonging to MoSe_2_ in the diffractograms, thus we can state that the nanomaterials are free of any impurities. The (002) peak arises due to the stacking of the layers in multilayer nanosheets. The appearance of the (002) peak in both diffraction patterns suggests the formation of multilayer MoSe_2_ [18]. However, the (002) diffraction peak in the MoSe_2_ nanosheets seems much broader than the (002) diffraction peak of the MoSe_2_ nanoflowers. This broadening is attributed to an inhomogeneity in the spacing between the van der Waals planes [24]. The Raman spectrum of MoSe_2_ has two main characteristic peaks which are the out of plane A_1g_ and the in plane E^1^_2g_, the mono or few-layer nanomaterials are identified by the shift in position of these characteristic peaks from their positions in the bulk material [25]. The Raman spectra of MoSe_2_ nanosheets and MoSe_2_ nanoflowers are shown in Figure 2.

The appearance of the characteristic out of plane A_1g_ and in plane E^1^_2g_ bands in both the Raman spectra confirms the formation of MoSe_2_. The A_1g_ appears at 242 cm^−1^ and the E^1^_2g_ appears at 286 cm^−1^ in the bulk [25]. The Raman spectra for both the nanosheets and nanoflowers show the A_1g_ has red-shifted from the bulk to 237 cm^−1^. The E^1^_2g_ has shifted to 285 cm^−1^. The red-shift of both the A_1g_ and E^1^_2g_ is indicative of the formation of few-layers [26]. There is one other characteristic peak observed in both the Raman spectra, the B^1^_2g_ which has been shown to not appear in monolayers or in the bulk materials. This peak only appears for few-layer materials with a layer number between 2–5 layers [25]. This result is in agreement with the PXRD analysis which confirmed the formation of few-layers nanosheets by the appearance of the (002) diffraction peak. The peak positions of the two samples do not show a significant difference from one another so the number of layers in the two samples is comparable. 

**Figure 3:**
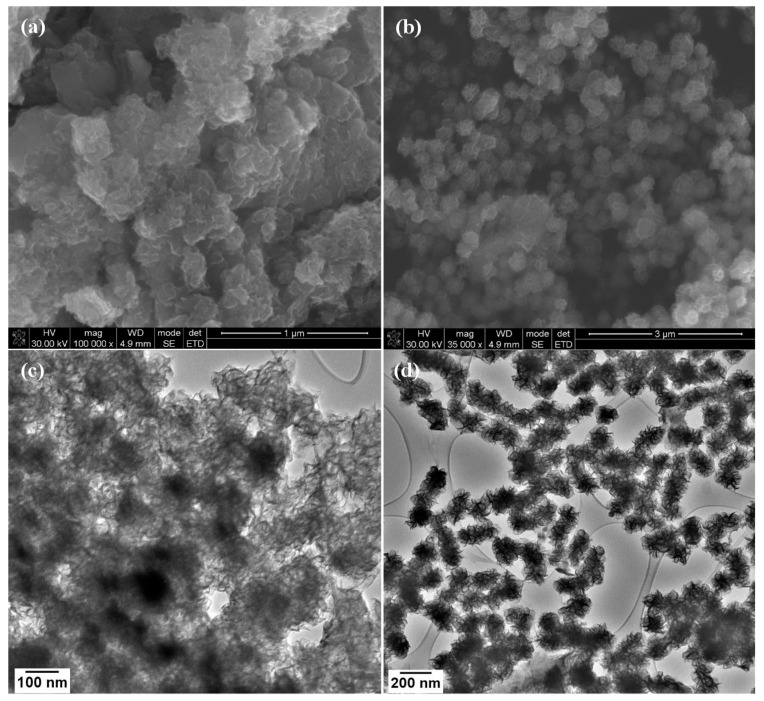
SEM images of (**a**) MoSe_2_ nanosheets and (**b**) MoSe_2_ nanoflowers and TEM images of (**c**) MoSe_2_ nanosheets and (**d**) MoSe_2_ nanoflowers.

**Figure 4:**
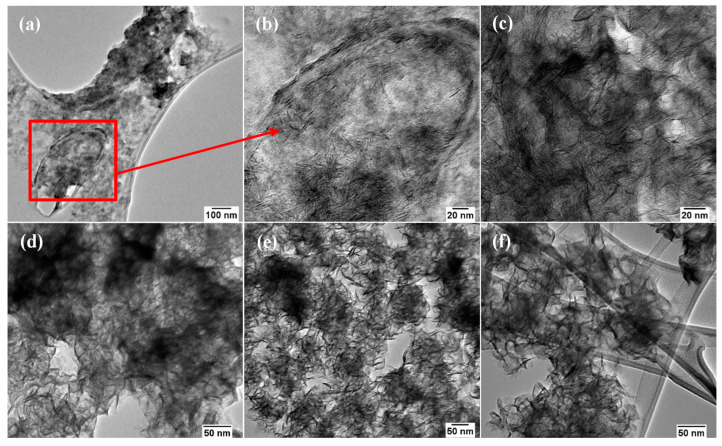
The TEM images of the MoSe_2_ nanosheets: (**a**–**c**) are images of the nanomaterial taken at 30 min, (**d**–**f**) are the TEM images for MoSe_2_ wrinkled few-layer nanosheets at 60, 90 and 120 min.

The images in Figure 3a,c show a network of interconnected wrinkled few-layer nanosheets, while the images in Figure 3b,d show the formation of MoSe_2_ nanoflowers which are composed of small lateral dimension nanosheets that seem to form from one central point. To investigate the formation of the wrinkled few-layer nanosheets a time study of the reaction was conducted where aliquots were obtained at the 30, 60, 90, and 120 min time intervals. Initial observation of the 30 min sample under low-resolution TEM revealed the appearance of a flocculate. This flocculate has also been observed by Savjani et al. in the synthesis of MoSe_2_. The flocculates have lateral dimensions of several hundred nanometres. They seem to mould and blanket the lacey carbon grids as shown in Figure 4a [27]. A closer look at these flocculates at higher magnification revealed that some ultrathin MoSe_2_ nanosheets had begun to form Figure 4b. These nanosheets seem to overlap with one another forming an interconnected network of few-layer nanosheets Figure 4c. The HRTEM images in Figure S2 confirms these as MoSe_2_ nanosheets due to the lattice fringes being ~0.66 nm which is the d-spacing of the (002) lattice plane in multi-layer nanosheets.

Figure 4d shows that there is a transition that occurs between 30 and 60 min that results in a change of the morphology of the nanosheets. At 30 min flocculates of the material with small ultrathin nanosheets in an OAm matrix can be observed. As the reaction continues to run for a longer period the flocculate is gradually phased out to form a network of densely packed wrinkled few-layer nanosheets beginning at 60 min as shown in Figure 4d. This transition continues from 60 to 120 min as shown in Figure 4e,f. This transition suggests that the reaction had not run to completion at 30 min, this could be due to the slow release of the Mo monomer as the Mo(CO)_6_ decomposes via decarbonylation of the CO ligands [28]. The formation of MoSe_2_ via Mo(CO)_6_ precursor is shown in Equations (1) and (2):

(1) $$2{\mathrm{CH}}_{4}N_{2}\mathrm{Se}\;\to 2H_{2}\mathrm{Se}+2C{\left(\mathrm{NH}\right)}_{2}$$

(2) $$\mathrm{Mo}{\left(\mathrm{CO}\right)}_{6}+2H_{2}\mathrm{Se}\;\to {\mathrm{MoSe}}_{2}+6\mathrm{CO}+2H_{2}$$

**Scheme 1:**
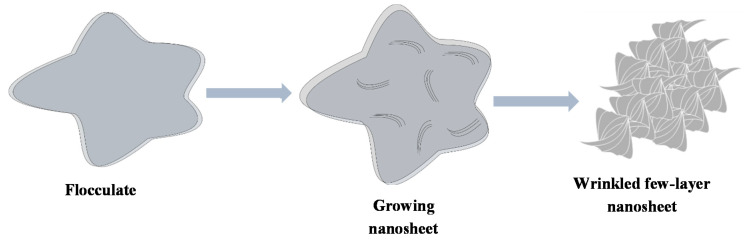
Mechanism for the formation of few-layer nanosheets.

The amorphous flocculate which is made up of the monomer from the decomposed precursors is consumed as the reaction runs for longer time periods until the large area wrinkled few-layer nanosheets are formed; a schematic diagram of the process is depicted in Scheme 1. 

This phenomenon was also observed by Gao and co-workers [29], who were able to form few layer nanosheets from the flocculate by increasing the reaction temperature in their microwave synthesis reaction when synthesizing MoS_2_ nanosheets. They suggested that the few layer nanosheets are formed by consuming the amorphous material in the flocculates as suggested in the process described in this synthetic method. However, in this work it has been proven that the few layer nanosheets form and grow from the flocculates at 300 °C just by increasing the reaction time from 30 to 60 min, which suggests that the temperature at which the reaction occurs is suitable for the synthesis of the material but longer reaction times are needed for the reaction to run to completion.

**Figure 5:**
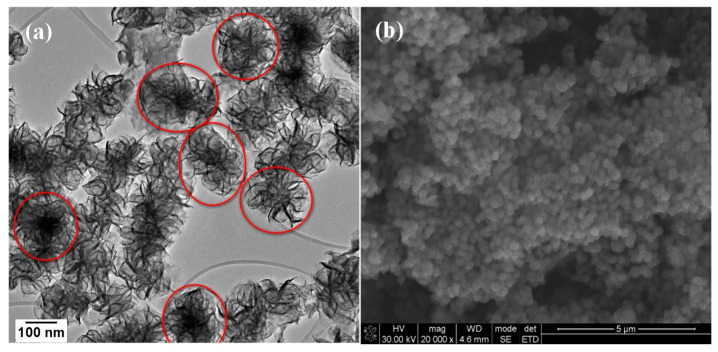
(**a**) TEM and (**b**) SEM images of MoSe_2_ nanoflowers formed at 30 min reaction time.

**Scheme 2:**
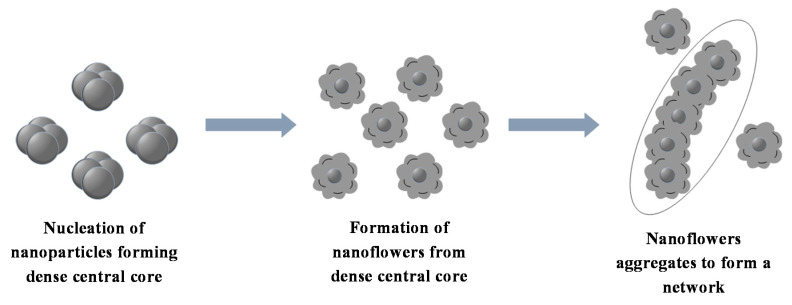
Mechanism for the formation of MoSe_2_ nanoflowers.

To elucidate the formation mechanism for the nanoflowers a time study was also done. However, the nanoflowers had already formed 30 min into the reaction hence only the 30 min sample is shown. The TEM and SEM images are shown in Figure 5a,b, respectively. The synthesis procedure for MoSe_2_ nanoflowers was slightly modified from the method used to synthesize the wrinkled few-layer nanosheets by changing the metal precursor from molybdenum hexacarbonyl to molybdic acid. It was found that the use of molybdic acid (H_2_MoO_4_) resulted in the formation of a network of nanoflower-like nanomaterials that arise through the formation of individual nanoflowers that are formed out of a central core as opposed to the flocculate observed when molybdenum hexacarbonyl was used [30]. Nanosheets with small lateral dimensions grow randomly outward from this central core in a fashion closely resembling a blooming flower. However, the nanoflowers that form do come together and agglomerated forming an interconnected network of these nanoflower structures. A schematic representation of the process is shown in Scheme 2.

The mechanism by which the dense central cores form can be understood by considering the decomposition of molybdic acid in oleylamine. The product obtained when molybdic acid is heated to 300 °C in oleylamine was found to be a mixture of MoO_4_^2−^ and oleylamine; this was confirmed by FTIR shown in Figure S3 [30]. The FTIR spectra show the characteristic antisymmetric stretching vibration of Mo-O-Mo in MoO_4_^2−^ at 827 cm^−1^ [31]. The anion has been shown to react with S^2−^ in the synthesis of MoS_2_ to form the intermediate MoO_4_^2−^ which converts to the MoS_2_ as the Mo(VI) is reduced to Mo(IV) [32,33]. A similar phenomenon occurs in the formation of MoSe_2_. The MoO_4_^2−^ has been shown in this work to readily react with H_2_Se to form nanoflowers through the formation of a central nucleation core. This rapid formation of the central core then results in the formation of the nanoflowers with the nanosheets growing outwardly simulating the behaviour of a blooming flower. Following the production of H_2_Se from selenourea (Equation (1)), its reaction with MoO_4_^2−^ is shown in Equation (3):

(3) $${\mathrm{MoO}}_{4}^{2-}+4H^{+}+2H_{2}\mathrm{Se}\;\to {\mathrm{MoSe}}_{2}+4H_{2}O\;$$

Brunauer-Emmett-Teller (BET) surface area analysis was used to determine the surface area of the MoSe_2_ wrinkled few-layer nanosheets and the nanoflowers. The surface area of the MoSe_2_ nanosheets was determined to be 14.1 m^2^/g while the surface area of the MoSe_2_ nanoflowers was determined to be 36.4 m^2^/g. The surface area of the MoSe_2_ nanoflowers was found to be larger than that of the MoSe_2_-nanosheets. This was expected because nanoflower structures have been known to possess higher surface area and also be microporous in nature [34].

### 3.2. Electrochemical Characterization

**Figure 6:**
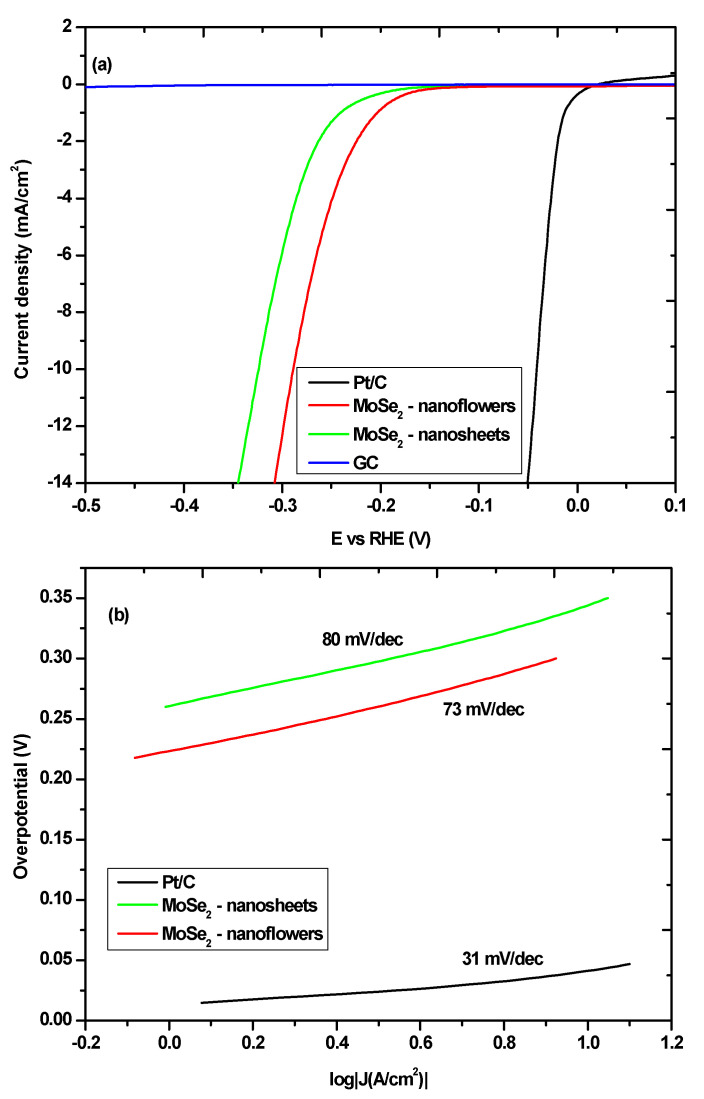
(**a**) Polarization curves for MoSe_2_ nanosheets and MoSe_2_ nanoflowers (**b**) Tafel slopes for MoSe_2_ nanosheets and MoSe_2_ nanoflowers.

Electrochemical characterization was done on the MoSe_2_ nanomaterials. This was done in order to show how the control of the morphology using the metal precursors can be used to improve the electrocatalytic activity of MoSe_2_ nanomaterials towards the hydrogen evolution reaction. Polarisation curves of the MoSe_2_ nanosheets and MoSe_2_ nanoflowers are shown in Figure 6a along with the polarization curves for 20% Pt/C and glassy carbon (GC). The 20% Pt/C was used as a standard and the GC polarization curve was done to ensure that the current response from the MoSe_2_ nanomaterials was not due to the effect of the GC. 

The polarization curves show that the nanoflowers have superior electrocatalytic activity towards the HER compared to nanosheets. The on-set potential for the -nanoflowers was lower than that of the nanosheets at 156 mV and 182 mV, respectively. This shows that a smaller overpotential is needed to start the reaction for the nanoflowers than the nanosheets. This trend is also observed when looking at the over-potential needed to reach a specified current density; the over-potentials at approximately 10 mA/cm^2^ (ƞ_10_) for the nanoflowers and nanosheets were found to be 301 mV and 340 mV respectively. As expected, the Pt/C sample displayed a lower on-set potential and potential at 10 mA/cm^2^. The GC electrode displayed negligible activity towards the HER. The Tafel plots of the MoSe_2_ nanomaterials and the Pt/C samples are shown in Figure 6b.

The Tafel plot was used to ascertain the Tafel slope which is a measure of the amount of over-potential needed to increase the reaction rate by a factor of 10. More accurately the Tafel slope is used to analyze the sensitivity of the current response to the applied potential. The Tafel slope for Pt/C was found to be 31 mV/dec which is roughly the number reported for this particular catalyst [35,36]. The Tafel slope for the MoSe_2_ nanoflowers was determined to be lower than that of the MoSe_2_ nanosheets at 73 mV/dec and 80 mV/dec respectively. This result shows that the activity of the MoSe_2_ nanomaterials is partly dependant on the morphology of the nanomaterials. This result is not surprising as it has been determined that the active sites for the HER in TMDs such as MoSe_2_ are the edge sites and as such the morphology that can effectively expose these edge sites would, therefore, have a better activity towards the HER. In this case the nanoflowers seem to be more effective at exposing these edge sites compared to the nanosheets as seen by the superior performance of the nanoflowers compared to the nanosheets. This is consistent with what has been reported in the literature with respect to how the nanoflowers morphology seems to be more ideal for the HER compared to other nanomaterial morphologies [36,37]. This can also be attributed to the higher surface area of the nanoflowers compared to the nanosheets which may afford the nanoflowers more available active sites.

**Figure 7:**
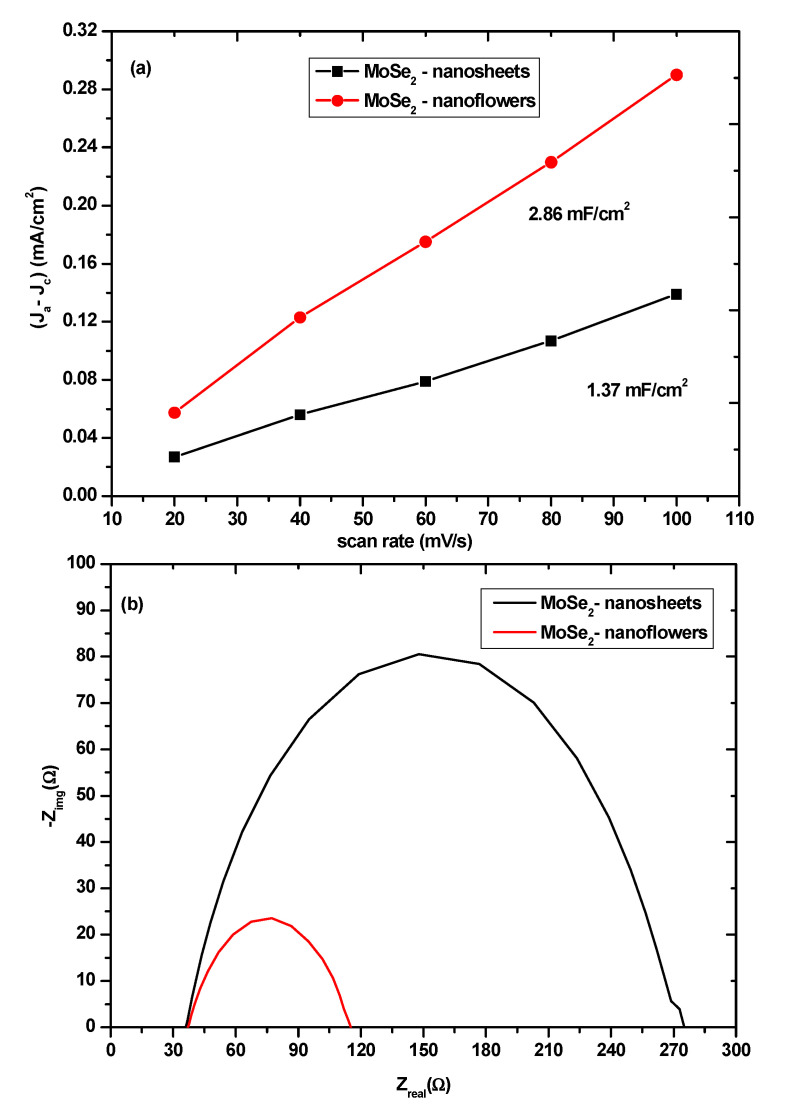
(**a**) linear fitting of the capacitive currents of MoSe_2_-nanosheets and MoSe_2_-nanoflowers vs the increasing scan rates. (**b**) Nyquist plots of the MoSe_2_-nanosheets and MoSe_2_-nanoflowers at −200 mV vs RHE.

To get a better understanding of the role of the surface area on the activity of the catalysts the C_dl_ plots for the two morphologies were obtained in order to get an estimation of the effective electrochemical surface area. Cyclic voltammetry (CV) was employed in order to determine the Cdl, the current response from the CV curves in the potential range between 0.2 V to 0.6 V shown in Figure S4 is due to the double layer charging. The electrochemical double layer can be used to estimate the effective electrochemical surface area because it is expected that the double layer capacitance is linearly proportional to the active surface area [38]. The estimation is done by plotting the difference between anodic and cathodic current densities (ΔJ = J_a_ − J_c_) vs the scan rate at 0.41 V vs RHE which is shown in Figure 7a. In this linear plot, the slope is the C_dl_. As expected, the C_dl_ of the MoSe_2_-nanoflowers was found to be higher than that of the MoSe_2_ nanosheets which indicates that the nanoflowers have more exposed edge sites. This would then explain why the nanoflowers seem to possess better electrocatalytic performance compared to the nanosheets.

The C_dl_ can be used to estimate the electrochemical surface area (ECSA); the ECSA is estimated using the ratio of the C_dl_ to the specific capacitance for the atomically smooth MoSe_2_ materials. By assuming that the specific capacitance for the atomically smooth MoSe_2_ is similar to that of MoS_2_ and using the Equation (4) [39].

(4) $$\mathrm{ECSA}=\frac{\mathrm{Cdl}\left(\mathrm{mF}/cm^{2}\right)}{\mathrm{Cdl}\left(\mu F/cm^{2}\right)}$$

**Figure 8:**
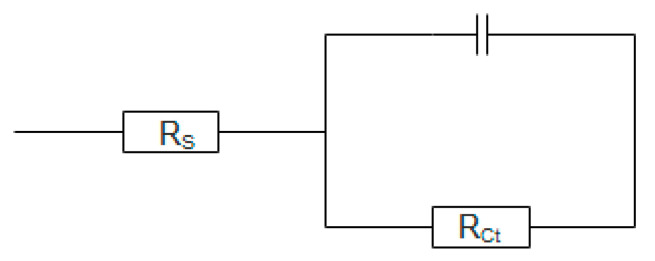
Equivalent circuit of the measured impedance spectra.

The ECSA of the nanoflowers and nanosheets was found to be 48 and 23, respectively. The higher ECSA of the nanoflowers further shows why the nanoflowers have superior electrochemical properties towards the HER. It should be noted that the wrinkled few-layer nanosheets also have a significant exposure of active edge sites because the nanosheets have a lot of standing edges but the nanoflowers have an advantage because all of the nanosheets that grow from the dense central-core terminate in the active edge sites. The electrode kinetics under HER conditions was investigated using impedance spectroscopy (EIS). The impedance spectra in Figure 7b can reveal information about the charge transfer resistance in the Nyquist plot. A small semicircle indicates a small charge transfer resistance which is the resistance associated with the transfer of electrons from the electrode material to the ions in solution. When the impedance data was fitted to the appropriate equivalent circuit shown in Figure 8, the values of the charge transfer resistance (R_ct_) were ascertained. 

The impedance spectra show that the charge transfer resistance of the MoSe_2_ nanoflowers (89 Ω) is much smaller than that of the MoSe_2_ nanosheets (220 Ω). This suggests that the electron transfer between the electrode material and the electrolyte in the nanoflowers is much more effective than in the nanosheets. Stability studies were also performed on the nanomaterials to determine whether they are stable in acidic media. An initial LSV scan was compared to a LSV scan that was measured after a thousand cycles were performed at 100 mV/s. As shown in Figure S5 there was no significant change in the LSV curves after a thousand cycles for both the nanoflowers and the nanosheets which suggest that the nanomaterials are fairly stable in acidic media. A comparison of the overpotential at 10 mA/cm^2^ and the Tafel slopes of MoSe_2_ synthesized using different techniques is shown in Table 1. The electrochemical characterisation for the data in the table was done at 0.5 M H_2_SO_4_. Table 1 shows that the overpotential and Tafel slope of the MoSe_2_ nanomaterials synthesized using the colloidal synthesis is comparable to those of other synthetic techniques. This is also true for the results found in this study.

## 4. Conclusions

In summary, a colloidal synthetic method was developed to synthesize a network of wrinkled few-layer nanosheets. The method was also adapted to synthesize MoSe_2_ nanoflowers by using molybdic acid as the metal precursor. The electrochemical properties of the MoSe_2_ nanosheets and MoSe_2_ nanoflowers were used to ascertain which morphology of the nanomaterials was most suitable for use as an electrode material for the HER. The overall electrochemical performance of the MoSe_2_ nanoflowers was determined to be superior to that of the MoSe_2_ nanosheets due to their higher effective electrochemical surface area. This indicates that the MoSe_2_ nanoflowers would be more suitable for use as HER electrode materials. 

## Figures and Tables

**Table 1:** Comparison of ƞ_10_ and Tafel slopes values of MoSe_2_ synthesized using different techniques.

HER Catalyst	Synthesis Technique	Ƞ_10_ (mV)	Tafel Slope (mV/dec)	Reference
**MoSe_2_/GC**	LPE	340	88	[21]
**MoSe_2_-nanoflowers**	CVD	220	61	[40]
**MoSe_2_-nanosheets**	Hydrothermal	305	69	[41]
**MoSe_2_-nanonetworks**	Colloidal	302	109	[42]
**MoSe_2_-nanoflowers**	Colloidal	233	89	[42]
**MoSe_2-x_-nanosheets**	Colloidal	300	98	[43]
**MoSe_2_-nanosheets**	Colloidal	340	80	This work
**MoSe_2_-nanoflowers**	Colloidal	301	73	This work

## Supplementary Materials

The following are available online at https://www.mdpi.com/2079-4991/10/9/1786/s1, Figure S1: TGA of selenourea showing the decomposition of the compound to H_2_Se and the carboamide (C(NH)_2_) at ~220 °C, Figure S2: HRTEM images of nanosheets synthesized at 30 min, showing the interlayer spacing of the nanosheets. The d-spacing of the lattice fringes can be used to determine whether the synthesized nanosheets are multi-layer or monolayer nanosheets. The d-spacing of the nanosheets was determined to be ~0.66 nm which is the d-spacing of the (002) lattice plane in multi-layer nanosheets. This confirms that the nanosheets at 30 min are indeed multi-layer, Figure S3: The FTIR spectrum of the product obtained when H_2_MoO_4_ is heated in oleylamine at 300 °C, Figure S4: CV curves of (a) MoSe_2_-nanosheets and (b) MoSe_2_-nanoflowers at scan-rates of 20, 40, 60, 80 and 100 mV/s, Figure S5: LSV curves of (a) MoSe2-nanoflowers and (b) MoSe2-nanosheets before and after a 1000 cycles of LSV.
