# Supplementary material for: Evaluating the Effect of Varying the Metal Precursor in the Colloidal Synthesis of MoSe2 Nanomaterials and Their Application as Electrodes in the Hydrogen Evolution Reaction
Source: Nanomaterials (Basel). 2020 Sep 9;10(9):1786. doi: 10.3390/nano10091786 (PMC7557751; doi:10.3390/nano10091786)
Supplement: Supplementary file 1 [file nanomaterials-10-01786-s001.pdf]

nanomaterials

Supplementary Materials

Evaluating the Effect of Varying the Metal Precursor in the Colloidal Synthesis of $MoSe_2$ Nanomaterials and Their Application as Electrodes in the Hydrogen Evolution Reaction

Zakhele Ndala [1], Ndivhuwo Shumbula [1], Siyabonga Nkabinde [1], Tshwarela Kolokoto [1], Obakeng Nchoe [1], Poslet Shumbula [2], Zikhona N. Tetana [1,3,4], Ella C. Linganiso [1,3,4], Siziwe S. Gqoba [1,*] and Nosipho Moloto [1,*]

1 Molecular Sciences Institute, School of Chemistry, University of the Witwatersrand, Private Bag 3, Wits, 2050, South Africa; 491384@students.wits.ac.za (Z.N.); 677753@students.wits.ac.za (N.S.); 564058@students.wits.ac.za (S.N.); 670850@students.wits.ac.za (T.K.); 2291563@students.wits.ac.za (O.N.); Zikhona.Tetana@wits.ac.za (Z.N.T.); Cebisa.Linganiso@wits.ac.za (E.C.L.)

2 Department of Chemistry, University of Limpopo Private Bag x1106, Sovenga 0727, South Africa; poslet.shumbula@ul.ac.za

3 DST/NRF Centre of Excellence in Strong Materials, University of the Witwatersrand, Private Bag 3, Wits, 2050, South Africa

4 Microscopy and Microanalysis Unit, University of the Witwatersrand, Private Bag 3, Johannesburg, Wits 2050, South Africa

* Correspondence: Siziwe.Gqoba@wits.ac.za (S.S.G.); Nosipho.Moloto@wits.ac.za (N.M.); Tel.: +2711-7176-774/+2711-7176-756 (S.S.G.); Fax: +2711-7176-749 (N.M.)

Supplementary Information

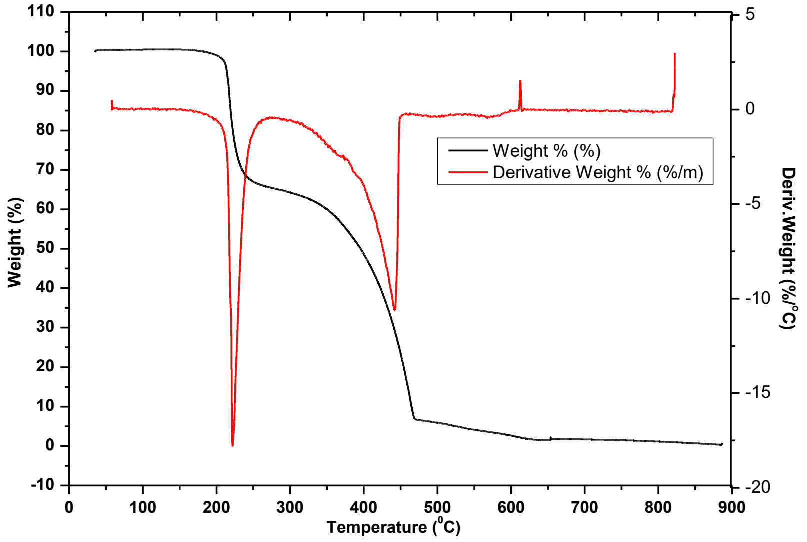

Figure S1. TGA of selenourea showing the decomposition of the compound to H_2Se and the carboamide ($C(NH)_2$) at ~220 °C.

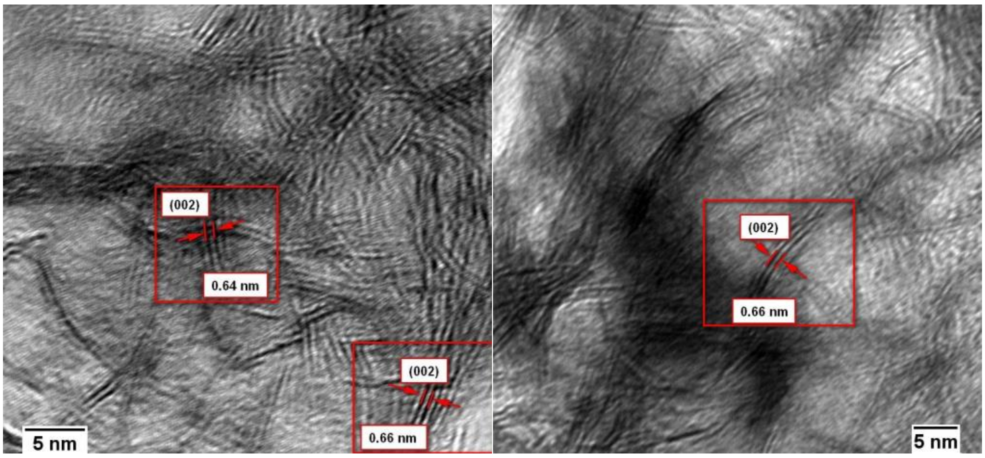

Figure S2. HRTEM images of nanosheets synthesized at 30 min, showing the interlayer spacing of the nanosheets. The d-spacing of the lattice fringes can be used to determine whether the synthesized nanosheets are multi-layer or monolayer nanosheets. The d-spacing of the nanosheets was determined to be ~0.66 nm which is the d-spacing of the (002) lattice plane in multi-layer nanosheets. This confirms that the nanosheets at 30 min are indeed multi-layer.

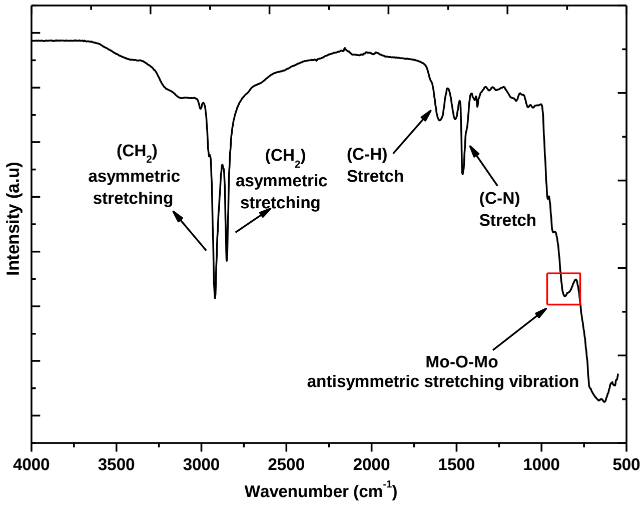

Figure S3. The FTIR spectrum of the product obtained when H_2MoO_4 is heated in oleylamine at 300 °C.

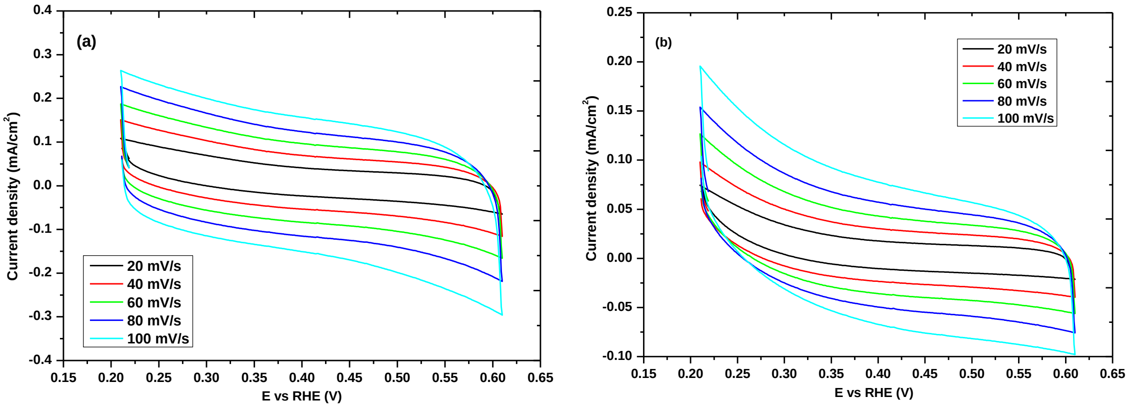

Figure S4. CV curves of (**a**) $MoSe_2$-nanosheets and (**b**) $MoSe_2$-nanoflowers at scan-rates of 20, 40, 60, 80 and 100 mV/s.

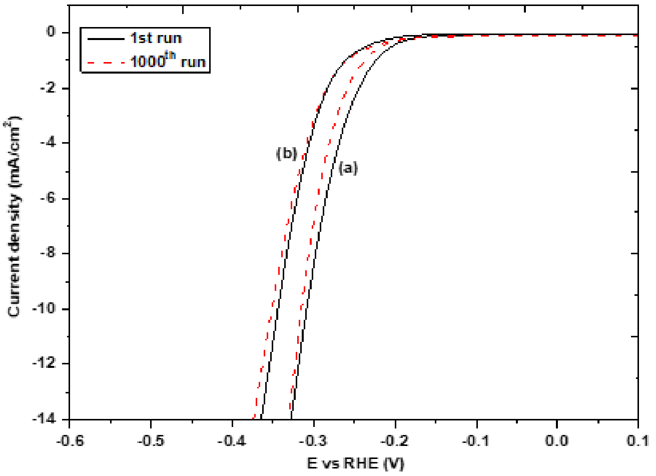

Figure S5. LSV curves of (**a**) $MoSe_2$-nanoflowers and (**b**) $MoSe_2$-nanosheets before and after a 1000 cycles of LSV.

© 2020 by the authors. Licensee MDPI, Basel, Switzerland. This article is an open access article distributed under the terms and conditions of the Creative Commons Attribution (CC BY) license (http://creativecommons.org/licenses/by/4.0/).
